# A cross-sectional follow up study to estimate seroprevalence of coronavirus disease 2019 in Kobe, Japan

**DOI:** 10.1097/MD.0000000000028066

**Published:** 2021-12-03

**Authors:** Asako Doi, Kentaro Iwata, Hirokazu Kuroda, Toshikazu Hasuike, Seiko Nasu, Hiroaki Nishioka, Keisuke Tomii, Takeshi Morimoto, Yasuki Kihara

**Affiliations:** a Department of Infectious Diseases, Kobe City Medical Center General Hospital, Kobe, Hyogo, Japan; b Division of Infectious Diseases Therapeutics, Kobe University Graduate School of Medicine, Kobe, Hyogo, Japan; c Department of Laboratory Medicine, Kobe City Medical Center General Hospital, Kobe, Hyogo, Japan; d Department of Respiratory Medicine, Kobe City Medical Center General Hospital, Kobe, Hyogo, Japan; e Department of Clinical Epidemiology, Hyogo Medical College, Nishinomiya, Hyogo, Japan; f Kobe City Medical Center General Hospital, Kobe, Hyogo, Japan.

**Keywords:** coronavirus disease 2019, Japan, seroprevalence

## Abstract

We conducted a study to estimate the seroprevalence of coronavirus disease 2019 (COVID-19) in Kobe, Japan with positive immunoglobulin G (IgG) rate of 3.3% (95% confidence interval [CI] 2.3%–4.6%) in April 2020. Because there were large concerns about the spread of COVID-19 among citizens thereafter, we conduct a follow-up cross-sectional study to estimate the seroprevalence, and we also added a validation study using a different assay.

We conducted cross-sectional serologic testing for severe acute respiratory syndrome coronavirus 2 (SARS-CoV-2) antibody using 1000 samples from patients at outpatient settings who visited the clinic from May 26 to June 7, 2020, stratified by the decade of age and sex. We used both Kurabo and Abbott serology assays to identify IgG against SARS-CoV-2.

There were 18 and 2 positive IgG among 1000 serum samples using Kurabo and Abbott serology assays, respectively (1.8%, 95% CI 1.1%–2.8%, and 0.2%, 95% CI 0.02%–0.7% respectively). By applying the latter figure to the census of Kobe City (population: 1,518,870), it is estimated that the number of people with positive IgG is 3038 (95% CI: 304–10,632) while a total of 285 patients were identified by polymerase chain reaction (PCR) testing at the end of the study period. Assuming Abbott assay as the reference, Kurabo assay had calculated sensitivity and specificity of 100% and 98.4% respectively. Age and sex adjusted prevalence of positivity was calculated to be 0.17%.

We found a lower seroprevalence than 2 months before in Kobe city although the figures were still higher than those detected by PCR. Kurabo assay showed more false positives than true positives despite reasonable sensitivity and specificity, due to low prevalence in Kobe.

## 1 Introduction

The government of Japan lifted the state of emergency declared over coronavirus disease 2019 (COVID-19) due to the lower number of newly diagnosed patients on May 25th.^[1]^ A total of 92,373 patients were diagnosed as COVID-19 with 1675 deaths as of October 20, 2020.^[2]^ In Kobe City, which is located in the middle of Japan, 1125 patients were identified with 15 deaths as of October 20, 2020.^[3]^ We conducted a cross-sectional study to estimate the seroprevalence of severe acute respiratory syndrome coronavirus 2 (SARS-CoV-2) infection in Kobe city in April 2020, since there was an upsurge of newly diagnosed cases then and Japan had restricted number of polymerase chain reaction (PCR) testing then, raising the concern that we might have been underestimating true epidemiology of the disease.^[4]^ In our previous study, we tried to estimate the number of people who have been infected with SARS-CoV-2 by using an immunoglobulin G (IgG) serology assay.^[5]^ It turned out 33 samples out of 1,000 specimens showed positive IgG, with seroprevalence of 3.3% (exact binomial confidence interval, 95% CI 2.3%–4.6%). However, serology assays have shortcomings of uncertain sensitivity and specificity, especially for the purpose of seroprevalence assay.^[6]^ Since serology assays with apparent better accuracy became available in Japan since our previous study period, we decided to conduct a follow-up study using 2 different serology assays to better estimate the seroprevalence of SARS-CoV-2 infection in Kobe city, to overcome some shortcomings of our previous study.

## 2 Methods

We conducted cross-sectional serologic testing for SARS-CoV-2 antibody (immunoglobulin G, or IgG) at Kobe City Medical Center General Hospital, a tertiary care medical center and designated hospital for COVID-19 in Kobe, Japan. As in our previous study, tests were done for randomly selected preserved serum from patients who visited outpatient clinics of the hospital and received blood testing for any reason. Patients who visited the emergency department or the designated fever consultation service were excluded to avoid the overestimation of SARS-CoV-2 infection.^[5]^ The serums of patients who visited the center from May 26 to June 7, 2020, were used for the analysis. Serums were preserved at −20°C and were defrosted upon testing.

Two serology assays were used to detect IgG against SARS-CoV-2. One, as in our previous study, was an immunochromatographic test by KURABO Industries Ltd (https://www.kurabo.co.jp/bio/English/index.html) and another was a chemiluminescent microparticle immunoassay (CMIA) (The SARS-CoV-2 IgG assay by Abbott, which was approved to use by the Food and Drug Administration (FDA) of the United States for emergency use (https://www.corelaboratory.abbott/us/en/offerings/segments/infectious-disease/sars-cov-2). Both assays target N protein of SARS-CoV-2. We used the latter as the reference to calculate the sensitivity and specificity of the former, since a previous study demonstrated its sensitivity and specificity of 100% at 17 days after symptom onset.^[7]^ However, a later evaluation found that the Abbott assay might have lower sensitivity at 14 days after symptom onset, with sensitivity and specificity of 93.9% and 99.6% respectively,^[8,9]^ we also calculated Clopper*-*Pearson exact CI using the numbers as a sensitivity analysis.^[10]^

For the immunochromatographic test, 10 microliters of serum were infused into the test kit, and the interpretation of the test results was made 15 minutes after the buffer was placed. On the other hand, the CMIA required 150 microliters of serum to infuse in the analyzer. Tests were conducted for 1000 samples, stratified by the decade of age and sex, with 50 samples taken from each stratum, and the same samples were used for both assays. If there were less than 50 samples from any stratum, additional samples were randomly obtained from other strata until all 1000 samples were completed.

The estimate of this study was a prevalence of seropositivity of IgG. The binominal test was used to estimate 95% CI. We used the McNemar test to assess the differences between the 2 assay methods. We compared the prevalence of the current study to that of the previous study by Chi-Squared test. As a sensitivity analysis, bootstrap procedures were conducted, using the basic percentile of the bootstrap distribution to construct CIs.^[11]^

Both unadjusted and age and sex adjusted estimations of the number of seropositive persons in Kobe city were calculated. For unadjusted estimation, we used Kobe City website for the population of Kobe City (of April 1, 2020 https://www.city.kobe.lg.jp/a89138/shise/toke/toukei/jinkou/index.html). For age and sex adjusted estimation, we used data of the national census conducted in 2015 (https://www.city.kobe.lg.jp/a73576/kenko/health/infection/protection/covid_19.html).

The ethics committee at Kobe City Medical Center General Hospital approved the current study. Because this study utilized the stored serum samples which were obtained in the non-research purpose practice, written informed consent was waived by the ethics committee and optout options for patients were provided at the homepage of our hospital, allowing them to refuse their serum to be used. We used the R software program, version 3.5.1 (R Foundation for Statistical Computing, Vienna, Austria) for all statistical analyses. The reported *P* values were two-tailed and *P* values less than .05 were considered statistically significant.

## 3 Results

There were 18 and 2 positive IgG among 1000 serum samples from the Kurabo immunochromatographic assay and Abbott CMIA respectively (1.8%, exact binominal 95% CI 1.1%–2.8%, and 0.2%, 95% CI 0.02%–0.7% respectively). 95% CI using bootstrap procedures were 0.9% to 2.6% and 0% to 0.4% respectively. Two samples were positive both with Kurabo and Abbott assays, but 16 samples had discordance in the results. The results of these 2 assays were statistically different (*P* < .0001). In addition, the result of the Kurabo assay at our current study was lower than our previous study significantly (1.8% vs 3.3%, *P* = .047). By applying the results of the Abbott assay to the population of Kobe city (population: 1,518,870), it is estimated that the number of people with positive IgG is 3038 (exact binominal 95% CI: 304–10,632, and bootstrap 95% CI 0–6075), the number 10.7 times higher than those identified by PCR by the end of the current study (285 patients. 1.07–37.3-fold for binominal 95% CI, and 0–21.3-fold for bootstrap 95% CI).

Calculated sensitivity and specificity of Kurabo assay using Abbott as the reference, were 100% and 98.4% respectively.

**Figure 1:**
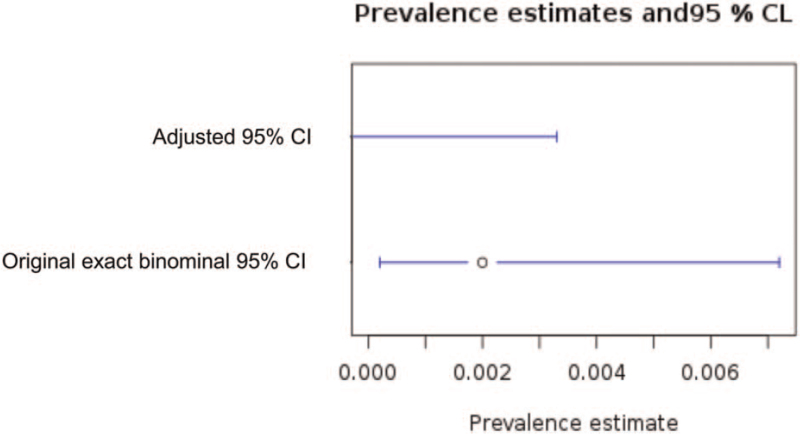
Estimated adjusted 95% CI of positivity of Abbott assay for SARS-CoV-2 using Clopper-Pearson exact CI. The upper bar denotes adjusted 95% CI and the lower bar is the original binominal 95% CI.

Assuming Abbott assay sensitivity and specificity of 93.9% and 99.6% respectively, the estimated Clopper*-*Pearson exact 95% CI was 0% to 0.3% respectively (Fig. 1).

Sample characteristics are shown in Table 1 stratified by the decade of age and sex of the patients. Table 2 shows the age and sex distribution of Kobe City at the national census held in 2015. The age and sex adjusted percentage of positivity of Kurabo and Abbott assay was 1.7% and 0.17% respectively.

**Table 1:** Sample characteristics.

Ages	Male	Test positive (Kurabo)	Test positive (Abbott)	Female	Test positive (Kurabo)	Test positive (Abbott)
Under 10-year-old	5	0	0	5	0	0
10–19	8	0	0	11	0	0
20–29	19	0	0	37	2	0
30–39	53	2	0	73	1	0
40–49	75	2	0	75	0	0
50–59	75	2	0	75	1	1
60–69	75	4	0	75	2	0
70–79	76	0	0	76	1	1
80–89	75	1	0	75	0	0
Over 90	18	0	0	19	0	0
Total	479	11	0	521	7	2

**Table 2:** Population of Kobe City based on 2015 census. Total number of the populations are aggregates of all age groups, which are different from what the census figure showed.

Ages	Male (%)	Female (%)
Under 10-year-old	61,242 (8.6)	58,671 (7.3)
10–19	70,275 (9.8)	67,661 (8.4)
20–29	73,973 (10.3)	78,787 (9.8)
30–39	87,806 (12.3)	95,510 (11.9)
40–49	109,303 (15.3)	116,372 (14.5)
50–59	89,500 (12.5)	98,220 (12.2)
60–69	105,160 (14.7)	114,649 (14.3)
70–79	77,705 (10.9)	96,228 (12.0)
80–89	36,428 (5.1)	61,344 (7.6)
Over 90	4475 (0.6)	15,169 (1.9)
Total	715,867	802,611

## 4 Discussion

Our results demonstrated that 1.8% and 0.2 of serum samples were positive for IgG against SARS-CoV-2, using Kurabo and Abbott serology assays respectively. In addition, seropositivity of Kurabo assay significantly decreased compared to our previous study, suggesting dwindling seropositivity over time.^[5]^ Our follow-up cross-sectional serological survey significantly decreased the estimated number of SARS-CoV-2 infected people in Kobe City by June 7, 2020. However, it still estimated a far number of infected than those identified PCR testing, and the majority of the infected people in Kobe city were likely to be undiagnosed. We still consider PCR testing is the best to identify persons with SARS-CoV-2 infection, but if it was not performed enough, as occurred in Japan, antibody tests could supplant it to better estimate the actual number of people infected retrospectively. Our results also demonstrated a significant difference between the 2 different assays. Assuming Abbott assay as the more accurate testing, most positive results provided by Kurabo assay are likely to be false positive, despite the fact that calculated sensitivity and specificity of Kurabo assay were high. This is most likely due to low prevalence, hence low pretest probability of the assay during the study period. If the pretest probability is very low, the positive test is likely to be false positive even if the test used had high specificity. The effectiveness of the Kurabo assay in a setting with high prevalence might be evaluated differently, like in the setting with the prevalence of 10% in New York in late March.^[12]^ Even Abbott assay yields false positive results more than true positive at a low prevalence condition.^[13]^

Seropositivity of Kurabo assay in our current study was significantly lower than the previous 1.^[5]^ Kurabo assay is a lateral flow immunochromatography testing, manually dropping serum to the kit and its results visually determined by an examiner, hence it is potentially liable to poor reproducibility. Abbot assay uses a system with mechanical sampling and automated interpretation, making these errors less likely to occur. The differences in the 2 assays could have occurred by these differences. A relatively short duration of the positivity of IgG could also have affected our results. One evaluation using the Abbott assay showed lower positivity at 21 days and 40 days after symptom onset, compared with ones conducted 14 days.^[8,9]^ Positivity of IgG decreased both in asymptomatic and symptomatic patients in the early convalescent phase,^[14]^ and another study found that IgG levels decreased in mild cases with a half-life of approximately 36 days.^[15]^ The same results were demonstrated in the subsequent systematic review.^[16]^ The same can occur in the Kurabo assay, suggested by our findings. These results raised a concern that seropositivity might not last as expected. The accurate estimation of seroprevalence of SARS-CoV-2 infection may be more difficult than we previously thought, and also raises another problem of the timing of the study to estimate the seroprevalence. With this potential reservation, the true seroprevalence of COVID-19 in Kobe city at its peak during the first phase of the epidemic could be higher than our estimates.

Our study has several inherent limitations. First, the true sensitivity and specificity of serology assays to estimate the prevalence of SARS-CoV-2 infection remain uncertain and there are inconsistencies among studies.^[17]^ Overestimates and underestimates of infections as discussed above could have occurred in our current study despite our multiple sensitivity analyses to better estimate the true seroprevalence of the infection. For example, a possibility of cross-reactivity with other conventional coronaviruses, such as the 1 causing common cold, must be considered to cause false positive results.^[18]^

Second, as pointed out in our previous study,^[5]^ the population of our cohort might not be the same as 1 in Kobe City. Although we adjusted the age and sex distribution to fit the general population, potential selection bias could be unavoidable.

Furthermore, some variants of concerns recently emerged might not react to the antibody testing than previous strains. Vaccination against the infection also may affect the usefulness of the antibody testing, although there were no significant variants or vaccination against COVID-19 during our study period.^[19,20]^ However, we used antibody tests for N protein, and most variants of concerns (VOCs) do have alteration in S protein instead. The targets of most vaccines are also S protein.^[21,22]^ Therefore, the antibody tests we used are less likely to be affected by both VOCs and vaccines. We need to keep evaluating the appropriate use of each test modality, such as PCR testing, antigen tests, antibody tests, or others, in the era of COVID-19 with VOCs and use of vaccines.

In conclusion, our cross-sectional serological study suggests that the actual number of people with SARS-CoV-2 infection in Kobe, Japan was estimated to be lower than our previous study, yet was more than the confirmed cases by PCR testing.

## Author contributions

**Conceptualization:** Asako Doi.

**Formal analysis:** Kentaro Iwata, Takeshi Morimoto.

**Funding acquisition:** Yasuki Kihara.

**Investigation:** Asako Doi, Hirokazu Kuroda, Toshikazu Hasuike.

**Methodology:** Kentaro Iwata, Seiko Nasu, Takeshi Morimoto.

**Project administration:** Yasuki Kihara.

**Supervision:** Kentaro Iwata, Hiroaki Nishioka, Keisuke Tomii, Takeshi Morimoto, Yasuki Kihara.

**Writing – original draft:** Asako Doi.

**Writing – review & editing:** Kentaro Iwata.
